# Alkaline Treatment Variables to Characterize Poly(Vinyl Alcohol)/Poly(Vinyl Butyral/Vinyl Alcohol) Blend Films

**DOI:** 10.3390/polym14183916

**Published:** 2022-09-19

**Authors:** Seong Baek Yang, Mohammad Rezaul Karim, Jungeon Lee, Jeong Hyun Yeum, Sabina Yeasmin

**Affiliations:** 1 Department of Biofibers and Biomaterials Science, Kyungpook National University, Daegu 41566, Korea; 2 Center of Excellence for Research in Engineering Materials, Deanship of Scientific Research, King Saud University, Riyadh 11421, Saudi Arabia; 3 The King Abdullah City for Atomic and Renewable Energy (K.A. CARE), Energy Research and Innovation Center, King Saud University, Riyadh 11451, Saudi Arabia

**Keywords:** poly(vinyl alcohol), poly(vinyl butyral), heterogeneous saponification, blend film

## Abstract

Novel poly(vinyl alcohol) (PVA)/poly(vinyl butyral–vinyl alcohol) (P(VB-VA)) films with improved hydrophobicity were prepared from poly(vinyl acetate) (PVAc)/poly(vinyl butyral) (PVB) blend films with various mass ratios by saponification in a heterogeneous medium. The successful conversion of PVAc to PVA and PVAc/PVB to PVA/P(VB-VA) films was confirmed by Fourier transform infrared spectrometry, X-ray diffraction, and proton nuclear magnetic resonance analysis. This study also shows that the degree of saponification (DS) depends on the saponification time. The maximum DS of 99.99% was obtained at 96 h of saponification for all films, and the presence of PVB did not affect the DS at saponification times of 48–96 h. The effects of the PVAc/PVB blend ratio before and after saponification were determined by contact angle measurement, and the hydrophobicity was found to increase in both cases with increasing PVB content. Additionally, all the films exhibited improved mechanical properties after saponification, and the treated films possessed an unusual porous and uneven surface, in contrast with the untreated films. The prepared films with improved hydrophobicity can be used for various applications, such as biomaterials, filters, and medical devices.

## 1. Introduction

Polymeric matrices are important in biomedicine, particularly for dialysis and cardiac treatment. The materials applied in biomedical devices must have certain characteristics, including low cost and biocompatibility [1]. Polymer blends have attracted considerable interest in academia and industry because blending makes it possible to develop materials with characteristics that are better than those of the component polymers [2,3,4]. These characteristics are affected by the miscibility of the constituents of the polymer mixture [5], which arises from interactions such as those resulting from dipole–dipole interactions and, in homopolymer mixtures, charge transfer complexes or segment–segment repulsion within blends [6].

**Figure 1:**
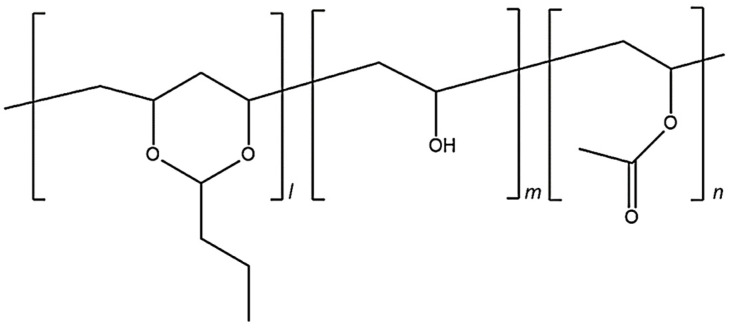
Chemical structure of poly(vinyl butyral) with the three different building blocks: vinyl alcohol (0.2); vinyl acetate (m < 0.01); and polyvinyl butyral (n~0.8).

Poly(vinyl butyral) (PVB) is thought to be an acetal, and is prepared by reacting an aldehyde and alcohol [7]. Figure 1 shows the structure of PVB, although it is typically not prepared in precisely this manner. As shown in Figure 1, it is a mixture of PVB, poly(vinyl alcohol) (PVA) and poly(vinyl acetate) (PVAc) segments. The polymer characteristics can be optimized by controlling the ratios of the three segments [8]. PVB reportedly exhibits high adhesion to glass, biocompatibility, and good solubility in alcohol; it has been applied in various fields, for example, as safety glass, coating films, and wound dressings [9,10,11,12,13,14].

Among various polymers, PVAc exhibits good mechanical stability, and PVAc films are easily prepared [15]. This synthetic polymer can be applied for various purposes, for example, detecting gas, moisture, and humidity. PVAc films are commonly used for oral drug delivery products as well as emission sensors for vehicles [16]. This type of polymer is expected to be applicable as an adhesive for porous materials, mainly wood, paper, bones, and cloth, and as a consolidation agent for sandstone [17].

PVAc has also been applied in medicine because of its biocompatibility. Because it is an inert polymer, it does not cause harmful reactions in living tissue [18,19]. PVA is a well-known water-soluble synthetic polymer for biomedical applications. Because of its solubility pattern and easy degradability, PVA is also considered to be a green polymer. It is compatible with various types of polymers, and it can be blended with various natural materials to extend its applicability.

PVA with improved properties can be prepared by heterogeneous saponification. For example, Yang et al. [20] prepared PVA/PVAc microspheres by heterogeneous surface saponification of PVAc microspheres and studied the degree of saponification (DS) in the presence of various nanoparticles. Lee et al. [21] also demonstrated a method of preparing syndiotactic PVA/poly(vinyl pivalate/vinyl acetate) microspheres using a heterogeneous saponification system, and examined the DS in terms of particle size and particle size distribution.

Our group has conducted the majority of the work on heterogeneous saponification. We have generated nanocomposite microspheres [22,23], nanofibers [24], films [25], and composite films [20]; in addition, several studies on heterogenous saponification are underway. PVA has unique characteristics, such as hydrophilicity, biodegradability, biocompatibility, ease of processing, and high cytocompatibility [26,27,28]. It does not irritate the skin and can adhere to soft tissues [29,30]. However, because of its high hydrophilicity and water solubility, the skin adhesion strength of PVA films is lower than that of commercial adhesives. Various techniques have been reported to improve the water resistance of PVA [31,32]. Our group recently prepared PVA/poly(methyl methacrylate–methallyl alcohol) (P(MMA–MAA)) hydrophobic–hydrophilic blend films from a PVAc/PMMA blend film by heterogeneous saponification, which proved to be a promising candidate for various medical applications, such as drug delivery and wound dressing [33].

Similarly, in this study, we prepared saponified PVA/PVB blend films from PVAc/PVB blend films using our unique saponification method. Due to the hydrophobicity of PVB and the hydrophilicity of PVA, these blend films are generally difficult to fabricate owing to the variations in the solubility of cosolvents, poor compatibility between the hydrophobic matrix and hydrophilic polymer, and phase separation may occur during the film production process [34,35]. The obtained saponified blend films had high surface areas, improved water resistance (maximum water contact angle (WCA), 53.53°), improved mechanical properties (maximum breaking stress, 33.98 MPA), and improved flexibility (maximum breaking strain, 16.99%), which are thought to be useful for various medical applications, including wound dressing. These results are reasonable compared to those of other reported PVA-based wound dressing materials [31,32,36,37,38]. Moreover, the prepared films with improved hydrophobicity can be used for various applications, such as in drug delivery [39], biomaterials [40], tissue engineering [41], filtration [42], food packaging [43], and medical devices [37].

## 2. Materials and Methods

### 2.1. Materials

Vinyl acetate (VAc) was purchased from Sigma-Aldrich (Seoul, Korea). First, it was cleaned using an aqueous NaHSO_4_ solution and distilled water. Next, anhydrous CaCl_2_ was used to dry the VAc. Finally, the VAc was distilled in a nitrogen atmosphere at reduced pressure. PVB (*M_w_* = 11,000) was purchased from Sigma-Aldrich. An initiator, 2,2′-azobis(2,4-dimethylvaleronitrile) (ADMVN), was acquired from Wako Co. (Tokyo, Japan); before use, it was recrystallized twice. PVA (*M_n_* = 127,000 g mol^−1^; DS = 88%) was also purchased from Sigma-Aldrich. The NaOH, Na_2_SO_4_, and methyl alcohol (MeOH) needed for the saponification reaction were obtained from Duksan Co. (Seoul, Korea). MeOH was used as a solvent to prepare the blend films.

### 2.2. Preparation of PVAc

PVAc powder was prepared by suspension polymerization as described in our previous publication [20]. In a chemical reactor, VAc was polymerized in order to prepare the PVAc resin suspension. A reactor (250 mL) was attached with a condenser, and a dispersing agent was dissolved by continuous mixing in a nitrogen environment. After degassing completion, the VAc monomer and the ADMVN initiator were introduced at 15 °C. Subsequently, the temperature was raised to 60 °C. The reaction mixture was left to stand for 1 day after a predetermined reaction time in order to isolate the globular PVAc particles.

### 2.3. Preparation of PVAc/PVB Blend Films and Heterogeneous Saponified PVA/P(VB-VA) Films

Film solutions of neat PVAc and PVAc/PVB with blend ratios of 90/10 and 80/20 were prepared successfully using methanol according to the solution weight. The polymer solution was kept at 10 wt. %. The prepared PVAc/PVB blend solution (10 g) was then cast in a Teflon Petri dish (100 mm × 10 mm). After casting, the Petri dish was placed in a vacuum oven at 35 °C to obtain a smooth surface. The prepared PVAc/PVB blend films were then converted to PVA/P(VB-VA) films by heterogenous saponification [25].

Heterogeneous saponification of PVAc/P(VB-VA) films was carried out in a flask equipped with a reflux condenser, a thermocouple, a dropping funnel, and a stirring device to synthesize PVA/P(VB-VA) nanocomposite films. In total, 10 g of sodium hydroxide, 10 g of sodium sulfate, 10 g of methanol, and 100 g of water was required to induce the saponification of the alkali solution. To prevent floating and twisting in the solution, rectangular pieces of each PVAc/PVB film (size varied depending on characterization method) were wrapped in mesh fabric by sewing. They were then placed into the flask comprising the alkali solution. After the saponification reaction, they were taken out of the flask, submerged in cold water, and left to precipitate for 1 min. After that, the saponified film was completely water washed. The saponified film was then thoroughly rinsed with water and dried at room temperature until completely dry. Except for NMR, where 0–96-h-saponified films are utilized, all characterization was conducted using film saponified for 72 h. PVB saponification is supported by another report, which showed the effect of alkaline treatment on PVB by NMR analysis [44].

### 2.4. Mechanical Properties of Films

The film thickness (Table 1) was calculated using a thickness gauge (Digital Vernier Caliper, Mitutoyo Korea Corporation, Daegu, Korea) at various points on each sample. Next, mechanical testing was performed using a universal testing machine (Instron 5567, Korea) according to ASTMD 638-96 type II requirements; the crosshead speed was 20 mm min^−1^. A fixed load (500 kgf) and displacement (200 mm) were used. For each condition, three samples (2 cm × 8 cm) were prepared, and the mean values of the three samples were calculated. The elongation and tensile strength were calculated according to the literature [45].

### 2.5. Surface Characterization of Films

The surface topography of the films was examined using optical microscopy (Olympus CKX41SF, Olympus Corporation, Tokyo, Japan), and the surface roughness was measured using atomic force microscopy (Park Systems NX20, Mannheim, Germany). The water contact angle (WCA) was measured according to the literature [46] using a contact angle meter (Dino-Lite, AM703MZT, Seoul, Korea).

### 2.6. Characteristics of Prepared Films

PVAc/PVB samples were prepared for proton nuclear magnetic resonance (^1^H-NMR) analysis (AVANCE III 500, Bruker, Germany) by dissolution in d_6_-dimethyl sulfoxide. After analysis, the DS was calculated from the ^1^H-NMR results according to the literature [25]. To confirm the conversion of the PVAc/PVB films to PVA/P(VB-VA) films, Fourier transform infrared (FTIR) spectroscopy was performed in a scan range of 400 to 4000 cm^−1^ at a resolution of 4 cm^−1^. To confirm that PVAc/PVB was converted to PVA/P(VB–VA), X-ray diffraction (XRD) analysis (D/Max–2500, Rigaku, Tokyo, Japan) was performed. During scanning, the pressure and voltage were 40 mPa and 40 kV, respectively, and the scanning speed was 4 °C min^−1^. Thermogravimetric analysis (TGA) (TA Instruments Q-50, USA) was performed to study the thermal properties of the saponified and unsaponified films. TGA was performed under a nitrogen atmosphere over a temperature range of 20–600 °C, at a heating rate of 10 °C min^−1^.

## 3. Results and Discussion

### 3.1. Optical Microscopy

**Figure 2:**
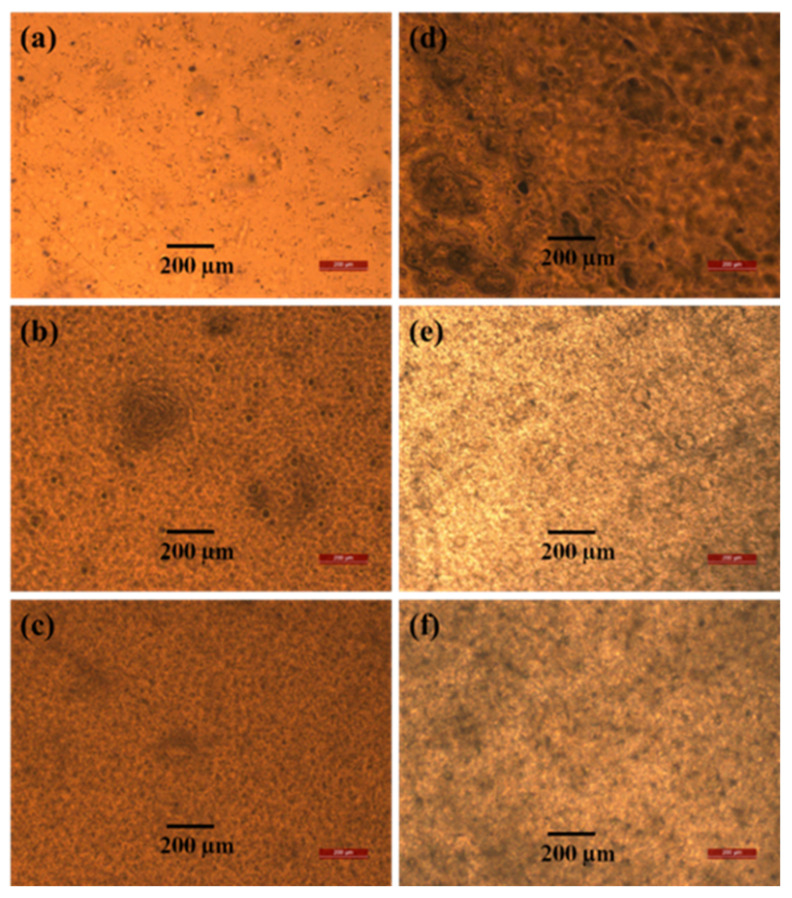
Optical microscopy images of unsaponified (**a**) PVAc film, (**b**) PVAc/PVB 90/10 film, and (**c**) PVAc/PVB 80/20 film and saponified (**d**) PVAc film, (**e**) PVAc/PVB 90/10 film, and (**f**) PVAc/PVB 80/20 film.

The optical microscopy images of the blend films in Figure 2 show that the PVAc matrix has a comparatively smooth surface, and the PVB particles are uniformly distributed (Figure 2a–c). Thus, there is strong interfacial adhesion between the two materials, which increases the tensile strength [47]. After saponification, voids appeared in the polymer matrix, and an unusual porous and uneven surface was obtained, which is expected to be useful for various applications because of the large surface area [24,25]. However, the tensile strength of all the saponified films does not decrease because PVAc is converted to PVA, which is confirmed by our results from other analyses (XRD, FTIR, and ^1^H-NMR measurements). At higher PVB contents, agglomeration occurred, which caused voids to form in the polymer matrix. These voids and agglomeration resulted in poor interfacial adhesion between the two components. As a result, the tensile strength decreased [48].

### 3.2. FTIR Spectroscopy

**Figure 3:**
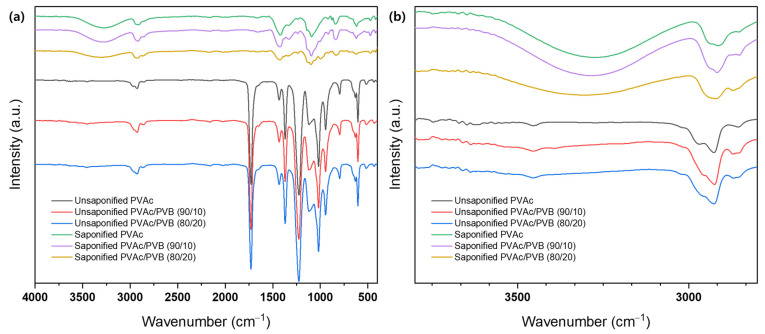
FTIR spectra of various film samples at (**a**) 4000−500 cm^−1^ and (**b**) 3500−3000 cm^−1^.

The structure of films with various blending ratios was evaluated using FTIR, as shown in Figure 3. The spectra of all samples show the typical transmission features of PVAc, as follows: C=O and C–O stretching between 1731 and 1733 cm^−1^ and other peaks at 1433–1435 cm^−1^ (C–H), 1371–1372 cm^−1^ (C–H), 1227–1235 cm^−1^ (C–O), and 1019–1021 cm^−1^ [49]. The spectra of the PVAc/PVB blend films exhibit the characteristic peaks of PVAc and PVB; the intensity of the characteristic peaks varies slightly with the weight ratio of the components. The intensity of all peaks around 600–1732 cm^−1^ decreases gradually with increasing PVB content in the blends; however, no distinct peak corresponding to PVB, for example, the –OH peak at 3486 cm^−1^, is identified, possibly because of the relatively low PVB content compared to PVAc in the PVAc/PVB 90/10 blend film. However, the vibration energy of –OH stretching is suppressed by steric hindrance in the 80/20 blend film, which may be attributed to the crosslinked network structure of PVB [50]. By contrast, all the films (pure PVAc/PVB and the 90/10 and 80/20 blends) show a significant change after saponification: broad bands appear at 3600–3200 cm^−1^ because of O–H stretching induced by both intermolecular and intramolecular hydrogen bonds [51]. 

The conversion of PVAc/PVB to PVA/P(VB–VA) caused the observed changes in vibration bands. Minor changes in peak position and intensity at 500–2000 cm^−1^ are also attributed to the formation of a PVA/P(VB–VA) film from a PVAc/PVB film. Figure 3b shows that as the PVB content in the PVAc/PVB blend film increased (PVAc/PVB 80/20), the O–H peak intensity decreased. The reason for this could be that both PVAc and PVB saponification were affected by steric hindrance at higher PVB contents because of the crosslinked network structure of PVB. Similar phenomena regarding the O–H peak intensity were reported in P(MMA–MAA) blend films [33].

### 3.3. XRD Analysis

**Figure 4:**
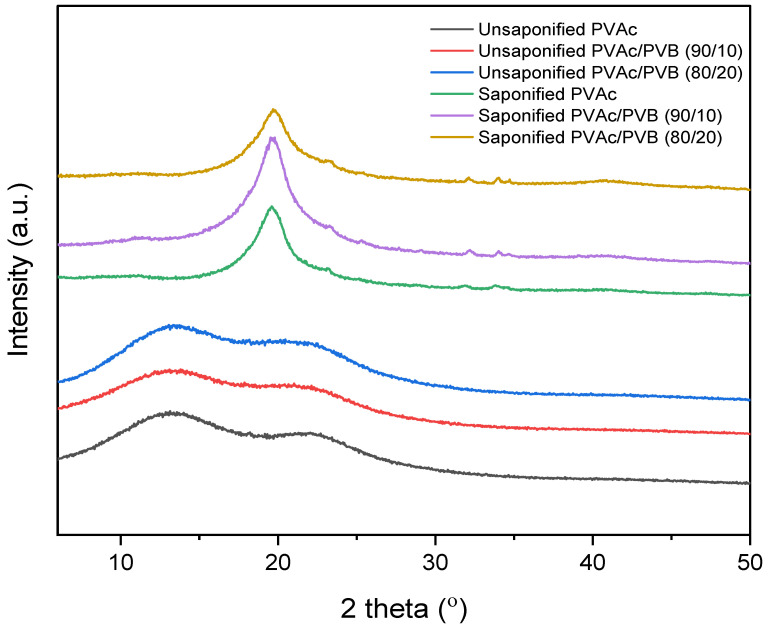
XRD patterns of unsaponified and saponified PVAc, PVAc/PVB 90/10, and PVAc/PVB 80/20 films.

The XRD patterns of the saponified and unsaponified films are shown in Figure 4. The PVAc sample shows the characteristic amorphous peaks at 2θ values of 13° and 22.5° [52]. The blend films show similar features. By contrast, the saponified PVAc sample shows a sharp crystalline peak at 2θ = 19.8°, which is characteristic of PVA and indicates that PVAc was converted to PVA [24]. Other blend film samples showed a similar trend; however, the diffraction peaks of saponified PVAc in the blend films are less intense at a higher saponified PVB content (the saponified PVAc/PVB 80/20 film), and the peak position is shifted slightly to lower values with increasing saponified PVB content. This result demonstrates that an increase in saponified PVB content disrupted the primary hydrogen bonds of PVA molecules and suppressed crystalline domain formation, thus decreasing the crystalline order of PVA. The major hydrogen bonding interaction among PVA and P(VB-VA) molecules may also have contributed to this phenomenon [53].

### 3.4. ^1^H-NMR Spectroscopy

**Figure 5:**
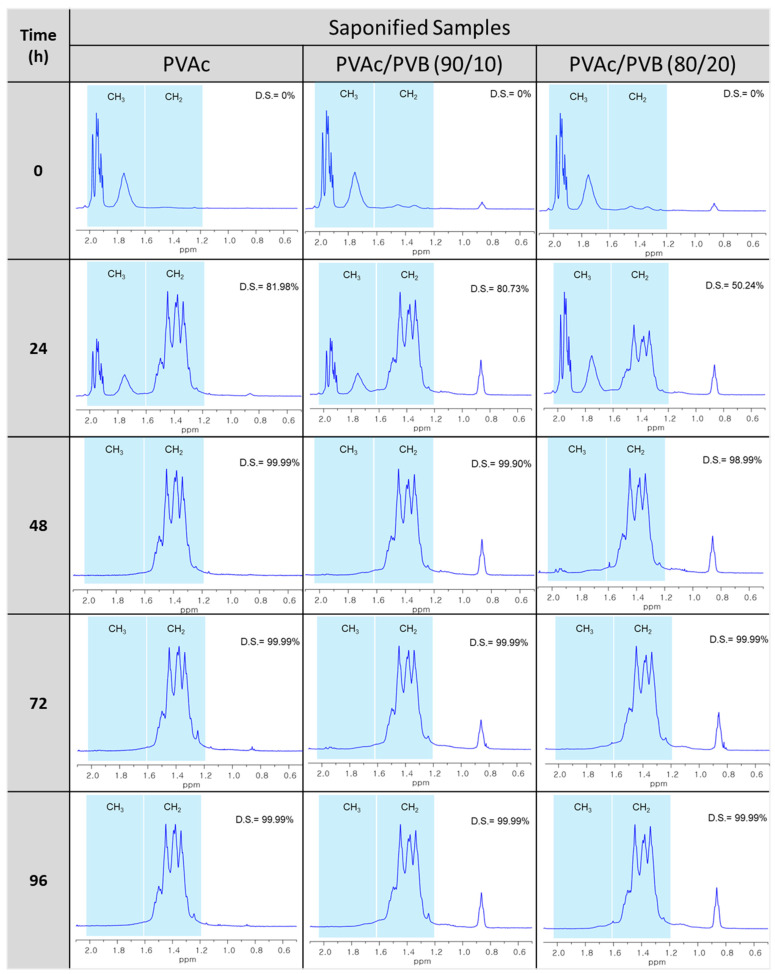
^1^H-NMR spectra of saponified PVAc, PVAc/PVB 90/10, and PVAc/PVB 80/20 films at a saponification temperature of 50 °C and various saponification times (0–96 h).

Figure 5 shows the ^1^H-NMR spectra of the unsaponified and saponified pure PVAc film and PVAc/PVB 90/10 and 80/20 blend films obtained at various saponification times. The spectra reveal the conformational changes of PVAc to PVA and PVAc/PVB to PVA/P(VB–VA), in good agreement with the FTIR results shown in Figure 3.

Table 2 shows the DSs of the PVA and blend films, which were estimated from the ratio of the areas of the methyl and methylene proton peaks (observed at 1.74 and 1.4 ppm, respectively) in the ^1^H-NMR spectra using the Mnova NMR program (AVANCE III 500, Bruker, Germany). At saponification times of 48, 72, and 96 h, the presence of PVB did not affect the DS of any of the films. For all the films, the methyl peak was clearly absent, and the methylene peak appeared after saponification, indicating the successful conversion of PVAc to PVA and PVAc/PVB to PVA/P(VB-VA) (Figure 5). That is, OCOCH_3_ in pure PVAc and the PVAc part of the PVB structure were converted to the OH group of PVA. At a saponification time of 24 h, the films were partially saponified. Interestingly, the CH_3_ peak of PVB at 0.9 ppm became more intense after saponification because the CH_3_ concentration increased after alkaline treatment of PVB, as the PVAc part of the PVB structure was also saponified [54,55].

### 3.5. Thermal Analysis

**Figure 6:**
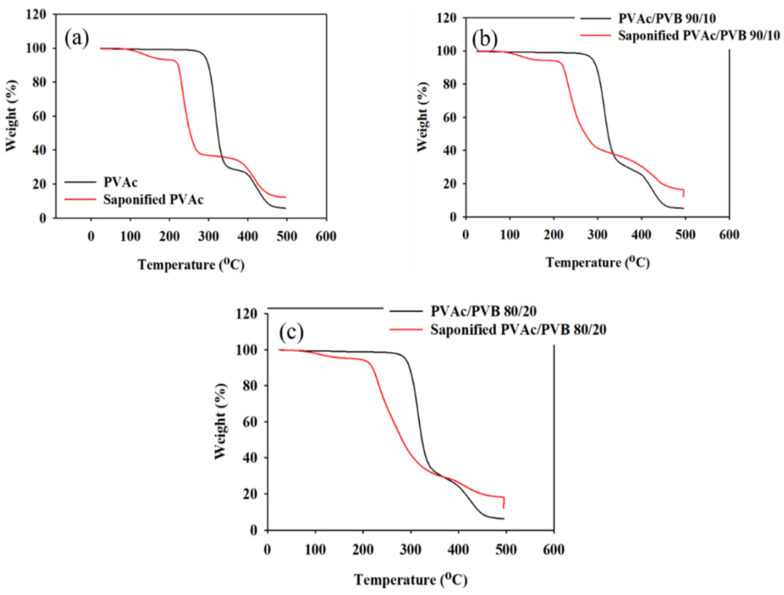
TGA data of unsaponified and saponified (**a**) PVAc film, (**b**) PVAc/PVB 90/10 blend film, and (**c**) PVAc/PVB 80/20 blend film (saponification temperature, 50 °C; saponification time, 72 h).

**Figure 7:**
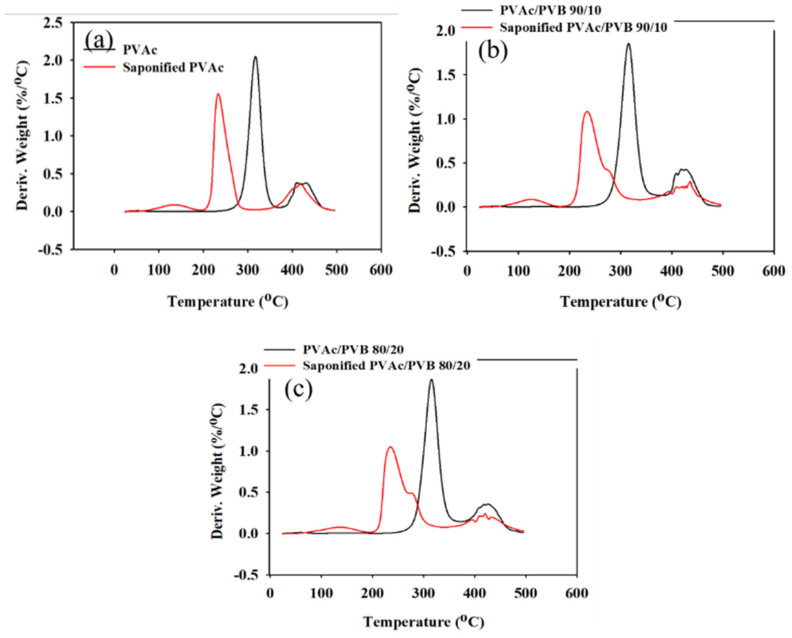
DTG data of unsaponified and saponified (**a**) PVAc film, (**b**) PVAc/PVB 90/10 blend film, and (**c**) PVAc/PVB 80/20 blend film (saponification temperature, 50 °C; saponification time, 72 h).

The effect of saponification on the thermal stability of the pure PVAc film and PVAc/PVB blend films with different ratios were examined by TGA and derivative thermogravimetry (DTG), and the results are shown in Figure 6 and Figure 7, respectively. The obtained degradation temperatures are listed in Table 3. As shown in Figure 6a, the saponified PVAc film showed higher thermal stability than the unsaponified PVAc film above the degradation temperature of 330.5 °C. The blend films exhibited similar behavior (Figure 6b,c).

The conversion of the methyl group of PVAc to the methylene group of PVA significantly decreased the thermal stability of the films. For the neat PVAc films, 10% weight loss occurred at approximately 296.85 °C; however, the value decreased to 219.45 °C after saponification. The PVAc/PVB 90/10 and 80/20 blend films exhibited similar behavior. In addition, the PVAc/PVB 80/20 films show slightly better thermal stability than the films with other blend ratios in terms of *T*_10%_ and *T*_max_, possibly because of the increase in water-insoluble polymer network structure with increasing PVB content.

For the neat PVAc films, *T*_max_ was 315.78 °C, and the maximum weight loss rate was 2.05% min^−1^. For the saponified PVAc, the values were 234.03 °C and 1.54% min^−1^, respectively. All the saponified film had similar *T*_max_ values.

As shown in Table 3, similar char yields were obtained for the pure PVAc (6.09%) and pure PVAc/PVB 90/10 films (5.04%), and the value increased for the pure 80/20 film (6.43%) because of the higher PVB content. By contrast, all the saponified film showed the same value (12.09%), possibly because the alkali-treated PVB does not affect the char yield as it was also converted to P(VB-VA).

### 3.6. Mechanical Properties

**Figure 8:**
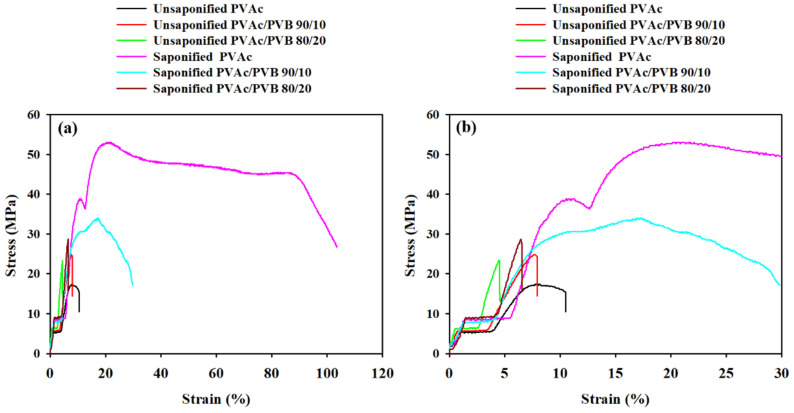
Stress–strain curves of film samples at strains of (**a**) 0 to 120% and (**b**) 0 to 30%.

Materials are expected to undergo different types of stresses during application; thus, it is necessary to measure the mechanical properties to predict the performance of materials. Figure 8 presents the characteristic stress–strain curves of the unsaponified and saponified films, and the mechanical properties are summarized in Table 4. The elongation at break (*ε*_b_), ultimate tensile strength (*σ*_u_), and tensile stress at break (*σ*_b_) of the treated and untreated PVAc/PVB blends and pure PVAc films were estimated. 

The stress–strain curve of the saponified pure PVAc film shows strain hardening and necking. Strain hardening indicates increased resistance to distortion after extension and the alignment of polymer chains in the saponified PVAc film; it is also affected by the presence of PVB in the saponified PVAc/PVB blend films. The same behavior was also observed in a PVA–polyethylene glycol–Ag_2_S hybrid polymer film [56].

Table 4 shows that the saponified PVAc film has the highest breaking stress (45.53 ± 0.25 MPa), which may be ascribed to the strain-induced alignment of PVAc polymer chain segments. Moreover, all the saponified films show higher breaking stress compared to the unsaponified films, possibly because the films were stronger after saponification. By contrast, all the saponified films had a higher breaking strain than the unsaponified films. Overall, saponification improved the mechanical properties of all the studied films. The reason may be the conversion of PVAc to PVA and PVAc/PVB to PVA/P(VB-VA) in the films, which is attributed to the crystalline structure of PVA; by contrast, PVAc is amorphous [57,58].

### 3.7. Contact Angle Measurements

To evaluate the wettability of the saponified and unsaponified films, the WCA was measured. As shown in Table 5, the pure PVAc film had a WCA of 57°, and the saponified PVAc film had a WCA of 27.41°; this difference results from the conversion of PVAc to PVA, which is hydrophilic. The WCA increased significantly with increasing PVB content in the PVAc/PVB blend films. The WCAs of nearly 70.54° and 76.54° for the PVAc/PVB 90/10 and PVAc/PVB 80/20 films indicate their high water resistance. A comparison with the contact angles of the pristine PVAc and PVAc/PVB blend films indicates that blending PVB with PVAc can enhance the water resistance of PVAc. The reason for this is that PVB itself is hydrophobic, although it is composed of multiple hydrophilic groups [59]. All the saponified films exhibit similar behavior; in addition, they are all more hydrophilic than their unsaponified counterparts. The increased hydrophilicity of the saponified films results from the conversion of PVAc and PVAc/PVB to PVA and PVA/P(VB–VA), respectively, as PVA is hydrophilic [60].

## 4. Conclusions

Saponified PVA and PVA/PVB films with blend ratios of 90/10 and 80/20 and various DSs (81.98–99.99%) were prepared from PVAc/PVB films at saponification times of 24, 48, 72, and 96 h at 50 °C. The conversion of PVAc/PVB to PVA/P(VB-VA) was confirmed by ^1^H-NMR. All the saponified films showed an unusual porous and uneven surface, which is expected to be useful for medical applications. Additionally, saponification improved the mechanical properties of all the studied films. The saponified PVAc film showed the maximum breaking stress (45.53 ± 0.25 MPa); however, the breaking stress was reduced by the presence of PVB in the saponified PVAc/PVB 90/10 and 80/20 blend films (to 33.98 ± 0.19 and 28.61 ± 0.16 MPa, respectively). Because the –OCOCH_3_ of PVAc was converted to the –OH of PVA, the thermal stability of the films was significantly lower after saponification; however, the saponified films showed greater thermal stability compared to the unsaponified films above the degradation temperature of 330.50 °C. The enhanced thermal properties of the prepared saponified PVA/PVB films were also revealed by the lower maximum weight loss rate (1.54 to 1.06% min^−1^) compared to unsaponified PVA/PVB films (2.05–1.87% min^−1^). The experimental results indicate that DS (81.98–99.99%) varies with reaction time under the same saponification conditions, and the detailed structure of PVA film can be established using XRD studies. The characteristic O-H peak at 2θ = 19.8° and broad bands appearing at 3600–3200 cm^−1^ in XRD pattern and FTIR spectra provide further evidence of the conversion of the PVAc/PVB to PVA/P(VB-VA). The WCA values of the prepared saponified PVA/PVB films (27.41–49.14°) compared to prepared unsaponified PVA/PVB films (57.68–76.54°) reveal that hydrophilicity increased after saponification because of the conversion of PVAc to PVA; however, the hydrophobicity increased with increasing PVB content. Finally, the saponified PVA and PVA/P(VB-VA) films with increased hydrophobicity show promise for applications in drug delivery, biomaterials, tissue engineering, filtration, food packaging, and medical devices.

## Figures and Tables

**Table 1:** Weight and thickness of saponified and unsaponified films.

Film Sample	Thickness (μm)	Weight (g)
Pure PVAc	140 ± 6	0.12 ± 0.08
Saponified Pure PVAc	150.4 ± 9.34	0.08 ± 0.10
PVAc/PVB 90/10	160 ± 5.09	0.12 ± 0.11
Saponified PVAc/PVB 90/10	213.2 ± 5.31	0.07 ± 0.10
PVAc/PVB 80/20	168 ± 5.83	0.18 ± 0.13
Saponified PVAc/PVB 80/20	230 ± 8.83	0.11 ± 0.10

**Table 2:** DS of samples for various saponification times.

Film Sample	24 h	48 h	72 h	96 h
PVAc	81.98%	99.9%	99.9%	99.99%
PVAc/PVB 90/10	80.73%	99.9%	99.9%	99.99%
PVAc/PVB 80/20	50.24%	98.99%	99.99%	99.99%

**Table 3:** TGA and DTG data of saponified and unsaponified PVAc films measured at a heating rate of 10 °C min^−1^. WLR_max_, maximum weight loss rate.

Film Sample	Char Yields (%)	*T*_3%_ (°C)	*T*_10%_ (°C)	*T*_max_ (°C)	WLR_max_ (% min^−1^)
PVAc	6.09	273.43	296.85	315.78	2.05
Saponified PVAc	12.09	122.98	219.45	234.03	1.54
PVAc/PVB 90/10	5.04	265.31	295.79	314.81	1.86
Saponified PVAc/PVB 90/10	12.09	139.48	220.80	233.48	1.09
PVAc/PVB 80/20	6.43	271.65	298.35	317.81	1.87
Saponified PVAc/PVB 80/20	12.09	134.48	222.15	234.83	1.06

**Table 4:** Breaking stress and breaking strain of saponified and unsaponified film samples.

Film Sample	Breaking Stress (MPa)	Breaking Strain (%)
PVAc	15.29 ± 0.13	10.63 ± 0.20
Saponified PVAc	45.53 ± 0.25	86.32 ± 8.60
PVAc/PVB 90/10	24.72 ± 0.15	7.58 ± 0.17
Saponified PVAc/PVB 90/10	33.98 ± 0.19	16.99 ± 0.23
PVAc/PVB 80/20	23.40 ± 0.13	4.29 ± 0.19
Saponified PVAc/PVB 80/20	28.61 ± 0.16	6.31 ± 0.17

**Table 5:** WCA of saponified and unsaponified film samples.

Unsaponified Film	Contact Angle (°)	Saponified Film	Contact Angle (°)
PVAc	57.68	PVAc	27.41
PVAc/PVB 90/10	70.54	PVAc/PVB 90/10	39.03
PVAc/PVB 80/20	76.54	PVAc/PVB 80/20	49.14
PVB	69.19	-	-

## References

[1] Di Landro L., Capone C., Inzoli F., Malacari P.E. (2005). Coextruded PVC tubes for biomedical application. J. Vinyl Addit. Technol..

[2] Taha T. (2019). Optical properties of PVC/Al_2_O_3_ nanocomposite films. Polym. Bull..

[3] Ahmed R. (2009). Optical study on poly (methyl methacrylate)/poly (vinyl acetate) blends. Int. J. Photoenergy.

[4] Nouh S., Benthami K., Abou Elfadl A., El-Nabarawy H.A. (2017). Modification induced by gamma irradiation in polystyrene/poly (methyl methacrylate) blends. Int. Polym. Processing.

[5] Ahmad J., Hågg M.B. (2013). Polyvinyl acetate/titanium dioxide nanocomposite membranes for gas separation. J. Membr. Sci..

[6] Botros S., Kenawy M., Younan A., El Kashef I. (2000). Thermal stability, swelling behaviour and dielectric properties of NBR/PVC-PVAc blends. Kautsch. Gummi Kunstst..

[7] McKeen L.W. (2016). Permeability Properties of Plastics and Elastomers.

[8] Haraya K., Hwang S.-T. (1992). Permeation of oxygen, argon and nitrogen through polymer membranes. J. Membr. Sci..

[9] Liu G.-S., Yan X., Yan F.-F., Chen F.-X., Hao L.-Y., Chen S.-J., Lou T., Ning X., Long Y.-Z. (2018). In situ electrospinning iodine-based fibrous meshes for antibacterial wound dressing. Nanoscale Res. Lett..

[10] Xu H., Li H., Chang J. (2013). Controlled drug release from a polymer matrix by patterned electrospun nanofibers with controllable hydrophobicity. J. Mater. Chem. B.

[11] Liu M.N., Yan X., You M.H., Fu J., Nie G.D., Yu M., Ning X., Wan Y., Long Y.Z. (2018). Reversible photochromic nanofibrous membranes with excellent water/windproof and breathable performance. J. Appl. Polym. Sci..

[12] Yan X., You M.-H., Lou T., Yu M., Zhang J.-C., Gong M.-G., Lv F.-Y., Huang Y.-Y., Long Y.-Z. (2016). Colorful hydrophobic poly (vinyl butyral)/cationic dye fibrous membranes via a colored solution electrospinning process. Nanoscale Res. Lett..

[13] Wu H., Zhang R., Sun Y., Lin D., Sun Z., Pan W., Downs P. (2008). Biomimetic nanofiber patterns with controlled wettability. Soft Matter.

[14] Chen S., Liu G.-S., He H.-W., Zhou C.-F., Yan X., Zhang J.-C. (2019). Physical structure induced hydrophobicity analyzed from electrospinning and coating polyvinyl butyral films. Adv. Condens. Matter Phys..

[15] Baskaran R., Selvasekarapandian S., Kuwata N., Kawamura J., Hattori T. (2007). Structure, thermal and transport properties of PVAc–LiClO4 solid polymer electrolytes. J. Phys. Chem. Solids.

[16] Veerabhadraiah A., Ramakrishna S., Angadi G., Venkatram M., Kanivebagilu Ananthapadmanabha V., Hebbale NarayanaRao N.M., Munishamaiah K. (2017). Development of polyvinyl acetate thin films by electrospinning for sensor applications. Appl. Nanosci..

[17] Petković G., Vukoje M., Bota J., Pasanec Preprotić S. (2019). Enhancement of Polyvinyl Acetate (PVAc) Adhesion Performance by SiO2 and TiO2 Nanoparticles. Coatings.

[18] Park K.R., Nho Y.C. (2004). Preparation and characterization by radiation of hydrogels of PVA and PVP containing Aloe vera. J. Appl. Polym. Sci..

[19] Andersen F. (1996). Amended final safety assessment of polyvinyl acetate. J. Am. Coll. Toxicol..

[20] Yang S.B., Park S.M., Kwon D.J., Shin J.-C., Sabina Y., Yeum J.H. (2020). Novel Poly (vinyl alcohol)/Clay Nanocomposite Film Prepared by the Heterogeneous Saponification of Poly (vinyl acetate)/Clay Nanocomposite Film. Sci. Adv. Mater..

[21] Lee S.G., Kim J.P., Lyoo W.S., Kwak J.W., Noh S.K., Park C.S., Kim J.H. (2005). Preparation of novel syndiotactic poly (vinyl alcohol) microspheres through the low-temperature suspension copolymerization of vinyl pivalate and vinyl acetate and heterogeneous saponification. J. Appl. Polym. Sci..

[22] Jung H.M., Lee E.M., Ji B.C., Deng Y., Yun J.D., Yeum J.H. (2007). Poly (vinyl acetate)/poly (vinyl alcohol)/montmorillonite nanocomposite microspheres prepared by suspension polymerization and saponification. Colloid Polym. Sci..

[23] Kwon I.J., Park S.M., Jeong M.G., Yang S.B., Yoo S.H., Jeong D.W., Sabina Y., Oh W., Choi H., Yeum J.H. (2016). Novel Poly (vinyl alcohol)/Carbon Nanotube Nanocomposite Microspheres Prepared via Suspension Polymerization and Saponification. J. Nanosci. Nanotechnol..

[24] Yang S.B., Lee H.J., Sabina Y., Kim J.W., Yeum J.H. (2016). Novel poly (vinyl alcohol) nanofibers prepared by heterogeneous saponification of electrospun poly (vinyl acetate). Colloids Surf. A.

[25] Yang S.B., Yoo S.H., Lee J.S., Kim J.W., Yeum J.H. (2017). Surface properties of a novel poly (vinyl alcohol) film prepared by heterogeneous saponification of poly (vinyl acetate) film. Polymers.

[26] Georgieva N., Bryaskova R., Tzoneva R. (2012). New Polyvinyl alcohol-based hybrid materials for biomedical application. Mater. Lett..

[27] Sulaiman N., Ghazali M., Majlis B., Yunas J., Razali M. (2015). Influence of Polyvinylalcohol on the Size of Calcium Ferrite Nanoparticles Synthesized Using a Sol-gel Technique. Proceedings of the International Conference for Innovation in Biomedical Engineering and Life Sciences.

[28] Chen X., Taguchi T. (2019). Hydrophobically modified poly (vinyl alcohol) s as antithrombogenic coating materials. Mater. Sci. Eng. C.

[29] Chaouat M., Le Visage C., Baille W.E., Escoubet B., Chaubet F., Mateescu M.A., Letourneur D. (2008). A novel cross-linked poly (vinyl alcohol)(PVA) for vascular grafts. Adv. Funct. Mater..

[30] Miyamoto A., Lee S., Cooray N.F., Lee S., Mori M., Matsuhisa N., Jin H., Yoda L., Yokota T., Itoh A. (2017). Inflammation-free, gas-permeable, lightweight, stretchable on-skin electronics with nanomeshes. Nat. Nanotechnol..

[31] Li H.-Z., Chen S.-C., Wang Y.-Z. (2014). Thermoplastic PVA/PLA Blends with Improved Processability and Hydrophobicity. Ind. Eng. Chem. Res..

[32] Huang Y., Zhao H., Chen S., Wan G., Miao D. (2022). Preparation of multifunctional wound dressings with composite PVA/PE films. J. Mater. Sci..

[33] Yang S.B., Jeong D.W., Lee J., Yeasmin S., Kim C.-K., Yeum J.H. (2022). Preparation of the Heterogeneous Saponified Poly (Vinyl Alcohol)/Poly (Methyl Methacrylate–Methallyl Alcohol) Blend Film. Materials.

[34] Li M., Li J., Zhou M., Xian Y., Shui Y., Wu M., Yao Y. (2020). Super-hydrophilic electrospun PVDF/PVA-blended nanofiber membrane for microfiltration with ultrahigh water flux. J. Appl. Polym. Sci..

[35] Sun K., Xia Y., Ouyang J. (2012). Improvement in the photovoltaic efficiency of polymer solar cells by treating the poly (3, 4-ethylenedioxythiophene): Poly (styrenesulfonate) buffer layer with co-solvents of hydrophilic organic solvents and hydrophobic 1, 2-dichlorobenzene. Sol. Energy Mater. Sol. Cells.

[36] Osorio M., Velásquez-Cock J., Restrepo L.M., Zuluaga R., Gañán P., Rojas O.J., Ortiz-Trujillo I., Castro C. (2017). Bioactive 3D-shaped wound dressings synthesized from bacterial cellulose: Effect on cell adhesion of polyvinyl alcohol integrated in situ. Int. J. Polym. Sci..

[37] Chen X., Taguchi T. (2020). Enhanced skin adhesive property of hydrophobically modified poly (vinyl alcohol) films. ACS Omega.

[38] Gao T., Jiang M., Liu X., You G., Wang W., Sun Z., Ma A., Chen J. (2019). Patterned Polyvinyl Alcohol Hydrogel Dressings with Stem Cells Seeded for Wound Healing. Polymers.

[39] Van Ngo H., Nguyen P.K., Van Vo T., Duan W., Tran V.-T., Tran P.H.-L., Tran T.T.-D. (2016). Hydrophilic-hydrophobic polymer blend for modulation of crystalline changes and molecular interactions in solid dispersion. Int. J. Pharm..

[40] Pearce A.K., O’Reilly R.K. (2021). Polymers for biomedical applications: The importance of hydrophobicity in directing biological interactions and application efficacy. Biomacromolecules.

[41] Zhang Y., Yuan Z.-P., Qin Y., Dai J., Zhang T. (2018). Comparative studies on hydrophilic and hydrophobic segments grafted poly (vinyl chloride). Chin. J. Polym. Sci..

[42] Sana S.S., Badineni V.R., Arla S.K., Boya V.K.N. (2020). Hydrophilic–hydrophobic polymer based blend membrane for separation of water–isopropanol mixtures by pervaporation. SN Appl. Sci..

[43] Ding J., Chen S.-C., Wang X.-L., Wang Y.-Z. (2009). Synthesis and properties of thermoplastic poly (vinyl alcohol)-graft-lactic acid copolymers. Ind. Eng. Chem. Res..

[44] Tupý M., Měřínská D., Svoboda P., Kalendová A., Klásek A., Zvoníček J. (2013). Effect of water and acid–base reactants on adhesive properties of various plasticized poly(vinyl butyral) sheets. J. Appl. Polym. Sci..

[45] Chiulan I., Frone A.N., Panaitescu D.M., Nicolae C.A., Trusca R. (2018). Surface properties, thermal, and mechanical characteristics of poly(vinyl alcohol)–starch-bacterial cellulose composite films. J. Appl. Polym. Sci..

[46] Yeasmin S., Yeum J.H., Yang S.B. (2020). Fabrication and characterization of pullulan-based nanocomposites reinforced with montmorillonite and tempo cellulose nanofibril. Carbohyd. Polym..

[47] Musa B.H., Hameed N.J. (2020). Study of the mechanical properties of polyvinyl alcohol/starch blends. Mater. Today.

[48] Karger-Kocsis J., Fakirov S. (2009). Nano- and Micromechanics of Polymer Blends and Composites.

[49] Triyana K., Rianjanu A., Nugroho D.B., As’ari A.H., Kusumaatmaja A., Roto R., Suryana R., Wasisto H.S. (2019). A highly sensitive safrole sensor based on polyvinyl acetate (PVAc) nanofiber-coated QCM. Sci. Rep..

[50] Muroga S., Takahashi Y., Hikima Y., Ata S., Ohshima M., Okazaki T., Hata K. (2021). New evaluation method for the curing degree of rubber and its nanocomposites using ATR-FTIR spectroscopy. Polym. Test..

[51] Yang S.B., Kim J.W., Yeum J.H. (2016). Effect of Saponification Condition on the Morphology and Diameter of the Electrospun Poly(vinyl acetate) Nanofibers for the Fabrication of Poly(vinyl alcohol) Nanofiber Mats. Polymers.

[52] Abdelghany A.M., Meikhail M.S., Asker N. (2019). Synthesis and structural-biological correlation of PVC\PVAc polymer blends. J. Mater. Res. Technol..

[53] Liao G.-M., Yang C.-C., Hu C.-C., Pai Y.-L., Lue S.J. (2015). Novel quaternized polyvinyl alcohol/quaternized chitosan nano-composite as an effective hydroxide-conducting electrolyte. J. Membr. Sci..

[54] Aruldass S., Mathivanan V., Mohamed A.R., Tye C.T. (2019). Factors affecting hydrolysis of polyvinyl acetate to polyvinyl alcohol. J. Environ. Chem. Eng..

[55] Qin X.-X., Cheng Z.-L. (2016). Application of ionic liquids as a catalyst in the synthesis of polyvinyl butyral (PVB) polymer. Chin. Chem. Lett..

[56] Devangamath S.S., Lobo B., Masti S.P., Narasagoudr S. (2021). Correction to: Thermal, mechanical, and AC electrical studies of PVA–PEG–Ag2S polymer hybrid material. J. Mater. Sci. Mater. Electron..

[57] Aziz S.B., Abdulwahid R.T., Rasheed M.A., Abdullah O.G., Ahmed H.M. (2017). Polymer Blending as a Novel Approach for Tuning the SPR Peaks of Silver Nanoparticles. Polymers.

[58] Čech Barabaszová K., Holešová S., Bílý M., Hundáková M. (2020). CuO and CuO/Vermiculite Based Nanoparticles in Antibacterial PVAc Nanocomposites. J. Inorg. Organomet. Polym. Mater..

[59] Peer P., Polaskova M., Musilova L. (2019). Superhydrophobic poly(vinyl butyral) nanofibrous membrane containing various silica nanoparticles. J. Text. Inst..

[60] Salam A., Khan M.Q., Hassan T., Hassan N., Nazir A., Hussain T., Azeem M., Kim I.S. (2020). In-vitro assessment of appropriate hydrophilic scaffolds by co-electrospinning of poly(1,4 cyclohexane isosorbide terephthalate)/polyvinyl alcohol. Sci. Rep..

